# Prediction of Structure of Human WNT-CRD (FZD) Complex for Computational Drug Repurposing

**DOI:** 10.1371/journal.pone.0054630

**Published:** 2013-01-25

**Authors:** Qurrat U. Ain, Umair Seemab, Sajid Rashid, Muhammad Sulaman Nawaz, Mohammad A. Kamal

**Affiliations:** 1 Department of Biosciences, COMSATS Institute of Information Technology, Islamabad Pakistan; 2 Korean Bioinformatics Center, Korea Research Institute of Bioscience and Biotechnology, Daejeon, Republic of Korea; 3 National Centre for Bioinformatics, Quaid-i-Azam University, Islamabad, Pakistan; 4 King Abdulaziz University, Jeddah, Saudi Arabia; 5 Unilever Centre for Molecular Informatics, Department of Chemistry, University of Cambridge, Cambridge, United Kingdom; Oak Ridge National Laboratory, United States of America

## Abstract

The observed genetic alterations of various extracellular and intracellular WNT (*Wingless*, *Int-1* proto-oncogene) signaling components can result in an increase or decrease in gene expression, and hence can be obstructed proficiently. These genetics target sites may include the prevention of WNT-FZD (Frizzled) binding, destruction of *β-catenin* and formation of *Axin*, *APC* and *GSK-3β* complex. Hence, the localized targeting of these interacting partners can help in devising novel inhibitors against WNT signaling. Our present study is an extension of our previous work, in which we proposed the co-regulated expression pattern of the WNT gene cluster (WNT-1, WNT-6, WNT-10A and WNT-10B) in human breast carcinoma. We present here the computationally modeled three dimensional structure of human WNT-1 in complex with the FZD-1 CRD (Cysteine Rich Domain) receptor. The dimeric cysteine-rich domain was found to fit into the evolutionarily conserved U-shaped groove of WNT protein. The two ends of the U- shaped cleft contain N-terminal and C-terminal hydrophobic residues, thus providing a strong hydrophobic moiety for the frizzled receptor and serving as the largest binding pocket for WNT-FZD interaction. Detailed structural analysis of this cleft revealed a maximum atomic distance of ∼28 Å at the surface, narrowing down to ∼17 Å and again increasing up to ∼27 Å at the bottom. Altogether, structural prediction analysis of WNT proteins was performed to reveal newer details about post-translational modification sites and to map the novel pharmacophore models for potent WNT inhibitors.

## Introduction

**Figure 1:**
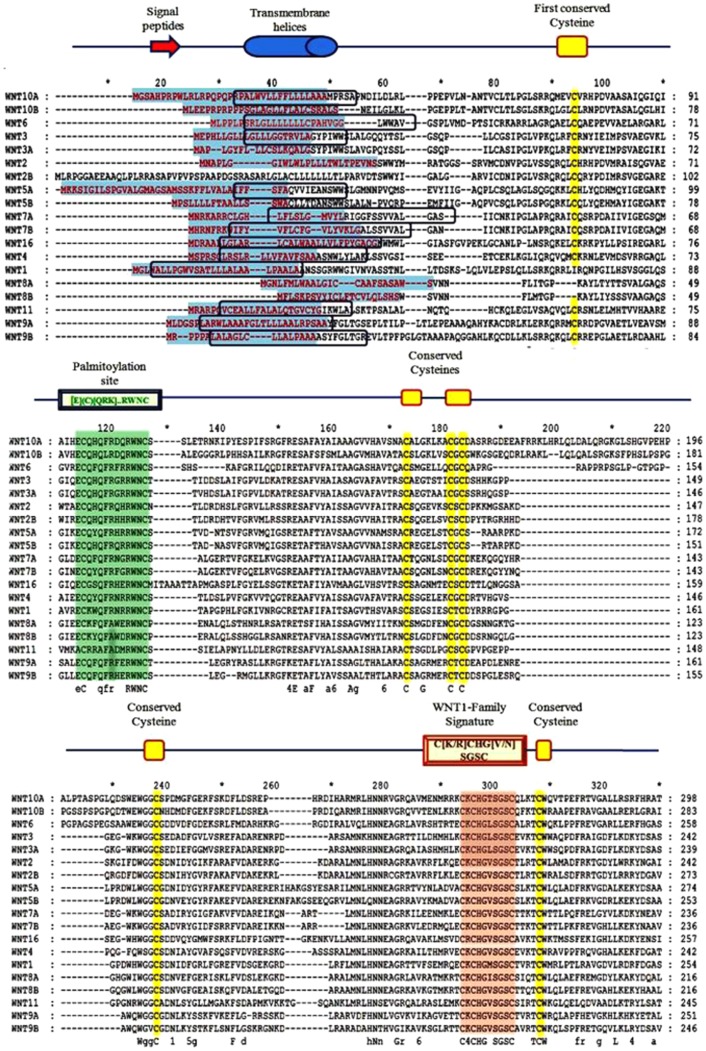
Multiple Sequence Alignment of 19 human WNTs paralogs. Signal peptide sequences are shown in red. Transmembrane helices of each WNT are shown in blue. The 23 conserved cysteines in all 19 WNT proteins are marked with yellow. These cysteine residues help in the formation of disulphide bridges and improve folding process. The palmitoylation site is also shown, displaying the conserved sequence for post-translational modification of WNTs. All WNTs have a common conserved family signature, as indicated. The signal peptide sequences were obtained from SignalP web server. TMHMM web server was used to determine helices inside, outside and between membranes. Motif enrichment analysis was performed using PRINTS, PRODOM, Blocks, Pfam and InterPro. The MSA was generated using ClustalX 2.0.

The large family of WNT ligands manipulates many diverse functions in humans, for example: embryonic induction, generation of cell polarity, and specification of cell fate [1]. At sequence-level, amino acid similarity within 19 WNT homologues ranges from 27% to 83% [2]. Approximately 43 kDa glycoprotein is encoded by WNTs [3]. The Wnt signal-transduction pathway has been widely conserved during animal evolution including mouse, Caenorhabditis elegans, and Drosophila [4], [5]. The conserved cysteine motifs at the C-terminus help WNT ligands to bind with Frizzled (FZD) receptors and initiate the WNT signaling cascade [1]. In summary, basic shared features of all WNTs comprise a signal sequence for secretion, the WNT family signature, a number of highly charged amino acid residues, numerous glycosylation sites, trans-membrane helices and conserved cysteines (**Fig. 1**).

The WNT signaling pathway is intricately linked with different types of cancers including colon cancer, breast cancer, gastric cancer, pancreatic and heptocellular carcinoma etc [6]–[8]. Tumor genesis can be caused by genetic alterations in *Axin*, *Adenomatous Polyposis Coli (APC)*, *β-catenin*; loss of *DKK*, *SFRP* & *WIF*; and mutations in β-catenin-Axin-adenomatous polyposis coli (APC)-glycogen synthase kinase (GSK)-3β multi-protein complex [9]. Combination of small secreted signaling proteins of the WNT family and their receptors, makes up the most complex relationship in this signaling pathway. WNT ligands bind at the Cysteine Rich Domain (CRD) of Frizzled receptors and together with FZD and LRP form a signaling complex to initiate the WNT pathway [10]. A number of routes have been observed to interrupt the WNT signaling pathway in numerous loss-of-function and gain-of-function mutations. These include the binding of WNT ligands with their receptors to form ligand-receptor complexes, extracellular inhibition of small WNT ligands by inhibiting their accurate processing, inactivation of the intracellular complex of *Axin* and *GSK-3β*, destruction of the destructor (*β-catenin*) and hyper-activation of naturally occurring WNT inhibitors (*WIF*, *DKK* and *SFRP*) [11]. Post-translational modifications are essential for accurate processing of WNT ligands. As WNTs are the secretary proteins, possessing a signal sequence which is necessary for proper targeting, this signal sequence is recognized by resident kinases of endoplasmic reticulum and hence glycosylate wingless proteins, before further processing [12]. WNT proteins are N-linked glycosylated, which may not be important for their activation but is necessary for their secretion and function. However, this glycosylation is always in competition with the disulphide bond formation. For the canonical WNT signaling pathway to become activated, palmitoylation of WNT is necessary [13]; on the other hand this palmitoylation also helps WIF to inhibit WNT signaling [14]. Inside the endoplasmic reticulum (ER), accurate processing of WNT requires porcupine, which also causes its palmitoylation thus interfering with disulphide bond formation and finishing the process of glycosylation.

The most intricate and least studied route of WNT signaling inhibition includes the targeting of small WNT ligands and the study of the ligand-receptor complex. The reason for this is that the primary amino acid sequence of WNT implies that they are soluble in water; however, the secreted WNTs are surprisingly hydrophobic and are associated with membranes. The hydrophobicity of WNTs is one of the reasons why no crystal structure of WNT has yet been identified [12]. Complex dimerization of FZD CRD is also important for signaling pathway activation [15], [16].The purposes of our study is to computationally model the tertiary structure of human WNT and FZD CRD proteins and suggest the important interacting residues of the receptor and the ligand involved in the activation of this pathway which could possibly be targeted to inhibit the interactions and hence stop the abnormal signaling pathway. The computationally modeled three-dimensional structures of WNT and CRD yielded good quality values when critically analyzed through various evaluation softwares. The Root Mean Square Deviation (RMSD) values calculated for Cα and the side chain also verified the quality assurance of modeled structures. The binding of a dimerized CRD domain into the potential binding pocket of WNT demonstrates two important facts: firstly, the CRD dimerization is necessary for initiation of WNT pathway; second, the structural details of WNT can be exploited to locate the significant residues of palmitoylation and glycosylation for targeted drug therapies. Our present study is an extension of Ain et al., 2011 [17], in which we proposed the co-regulated expression pattern of WNT gene cluster (WNT-1, WNT-6, WNT-10A and WNT-10B) in human breast carcinoma. The present study aims to computationally model the structures of human WNT-1 and its receptor protein CRD of Frizzled. Along with this, structures of WNT-6, WNT-10A, and WNT-10B are predicted and refined. Lipid modified Xenopus WNT-8 in complex with Mouse FZD-8 was crystallized by Janda et al., 2012 [18]. However, in our study we modeled the binding interactions of WNT-1 complex with FZD-1 in *Homo sapiens*.

## Methods

### Dataset collection

The sequence similarities of the two closest homolog clusters, (WNT-1 and WNT-6) and (WNT-10A and WNT-10B) is 40% and 60% respectively. ClustalX [19] was used for sequence alignment and further editing was performed using GenDoc. Sequences of all WNT proteins were extracted from Ensemble genome browser [20], and subjected to PSI-BLAST with a protein structure database [21] to find the best template. As no crystal structure of WNT was available, the closest homolog retrieved was the ligand-binding face of the semaphorins revealed by the high resolution crystal structure of SEMA4D protein (pdb id: 1OLZ), having 39% sequence homology with WNT-2B.

### Homology Modeling

The three-dimensional structure prediction of WNT-2B (PM0078113) was done by comparative homology modeling of target sequences using Modeller 9v8 [22]. Using WNT-2B as template, structures of WNT-1 (PM0078114), WNT-6 (PM0078115), WNT-10A (PM0078128) and WNT-10B (PM0078116) were predicted. The molecular model of these proteins was subjected to optimization. The overall geometry optimization was followed by energy minimization of retrieved structure using Chimera1.5.2 [23]. This took a total of 100 steps (step size 0.02 A) employing conjugate gradient method followed by protonation of wild type histidines using AMBER ff98 method. The generated models were ranked on the basis of the Modeler objective function, obtained after more than ∼2000 average iterations of a template structure with simulated annealing algorithm using Modeller9V8. Later each generated model was processed for quality assurance. One of the quality assurance parameters includes DOPE plot. The discrete optimized potential energy (DOPE) profile plot of each target structure was generated through Modeller 9v8 with reference to the template, and the stability of generated target model was tested.

### Structure Validation and Analysis

Predicted 3D models were verified by PROCHECK [24] and validation of stereo- chemical quality of a models was performed through WHATIF, ERRAT, PROCHECK, PDBSUM, Ramachandran Plot2.0 [25] and Verify3D. A Ramachandran plot was generated for each computationally predicted structure to testify the steric hindrances of protein residues, and the outlier errors were corrected accordingly using WinCoot [26]. Most of the structural mis-alignment errors in rotamers occurred due to side chain packaging and folding. These problematic rotamers were refined using Dunbrack rotamer library. The values for RMSD (Root mean square deviation), SDM (Structural deviation measure), B-factor and Q (quality)-factor were used to verify further the quality of the resulting structures of WNT proteins as well as receptor CRD using Chimera 1.5.2 (Table S1). The Q-score assessment was given by Krissinel E and Henrick K, 2004 [27] as,
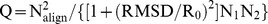
Where, N_1_ and N_2_ represent the number of residues of aligned structures. Q-finder and pocket-finder were used to determine the probable binding pockets of WNT protein. One of the important measures to calculate the geometry accuracy is RMSD, obtained after superimposition of the target to its native structure. RMSD depends on the number of equivalent atom pairs of both proteins (target and template) that are compared, which in turn depends upon the maximum allowed distance between atom pairs.

### Molecular Docking

In order to predict the exact amino acid residues involved in CRD dimmer interaction, docking studies were performed using AUTODOCK 4.2. Polar hydrogen atoms were added to both ligand and receptor molecules. A population size of 150 with 10 million energy evaluations was used. The number of total docking runs was set to 100. The grid size was to cover the whole receptor to find a potential binding pocket for the WNT-FZD complex. A hybrid Lamarckian genetic algorithm was used for flexible docking. Detailed docking and grid parameters are shown in Table 1. The other parameters were set to default values. The docking results were visualized and analyzed by using Chimera1.5.2 and VMD [28] tools. Protein- protein interactions were further verified using another web server called Patch dock [29]. Patchdock is a molecular docking server based on the principle of shape complementarity and its related server FireDock [30] and SymmDock were used for fine refinement of docked complex. Based on the atomic energy scores, the predicted protein binding was confirmed, refined and then used for results and analysis.

**Table 1:** List of grid and docking parameters used to perform docking experiment.

Grid Spacing	0.845 Å
Grid Center	78.45X, 54.33Y, 37.84Z
Translation step	2
Quatemion step	50
Torsion step	50
Torsional degrees of freedom	6
Rate of Gene Mutation	0.02
Rate of Crossover	0.8
Cluster Translation Step	2
Population Size	150
Max. number of evaluations	25000000
Global Free Energy	−33.37 kJ/mol
Atomic contact Energy	−4.49 kJ/mol

## Results

### Homology Modeling

Among 19 human WNT proteins, WNT-2B was taken initially to be modeled because of its greatest query coverage with the template (pdb ID: 1OLZ). Later, this structure was used as a template for other proteins. Each generated model was selected on the basis of modeler objective function. The lower the value of this objective function, the more preferred the model would be. Among 10 generated models of each protein, only one model (with least modeler objective function) was selected for further analysis.

### DOPE Plot

**Figure 2:**
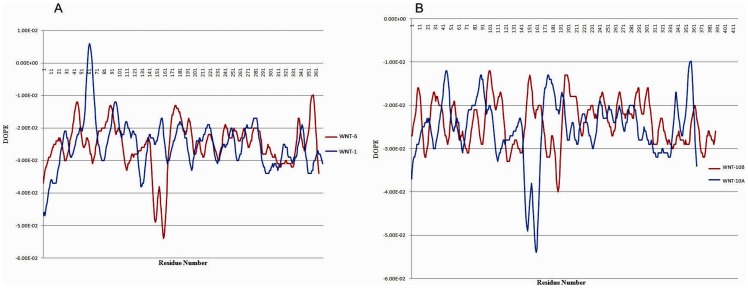
Evaluation of computationally modeled protein targets using DOPE plots. (A) DOPE profile plot of WNT-1 and WNT-6. The protein's residue numbers are plotted on the X-axis and the DOPE energies of each residue are plotted on the Y-axis. The structure of WNT-6 is modeled using WNT-1 as a template. The predicted model of WNT-6 has lower optimized energy than WNT-1. Lower energy confirms the higher stability of predicted model. **(B)** DOPE profile plot of WNT-10A and WNT-10B. The structure of WNT-10B is modeled using WNT-10A as a template. The predicted model of WNT-10B has lower optimized energy than WNT-10A. Lower energy confirms the higher stability of predicted model.

DOPE profiles of WNT predicted protein models were plotted against their residue numbers in **Fig. 2A** and **Fig. 2B**. Energy profiles of individual residues help in understanding the critical assessment areas, for example loop regions. Newly generated structures of proteins have shown low energy values as compared to their template residue energies.

### Q-Score

This scoring function tends to achieve a higher objective value as it is based solely on sequence similarity of target and template. Values of Q-score range from zero to one, where 1 represents the identical structures and 0 represent the dissimilar structures. The predicted models of CRD, WNT-1, WNT-6, WNT-10A and WNT-10B showed high Q-score.

### Structural Deviation Measure

The SDM of each protein should be lower when compared with its template protein. This confirms the good quality of the predicted structure. Some of the disturbing noise values in SDM were observed because of the sequence diversity between target and template proteins. However, the important functional domains and motifs showed 100% conservation. The quality parameters of modeled structures are defined in Table S1. The predicted 3D structure of the CRD complex after geometry optimization was also evaluated by RamachanranPlot2.0 which confirmed that all of the amino acid residues lay within the allowed region (except Ala 18 of beta chain as shown in Figure S1).

### Root Mean Square Deviation

**Figure 3:**
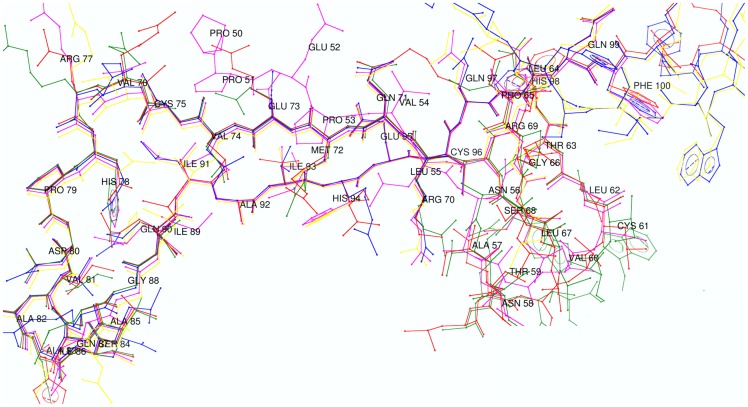
Superimposed 3D modeled structures of the (active conserved domain) WNT-1, -2B, -6, -10A and -10B. Highly conserved residues from 70–80 and 90 onwards are making the running backbone, whereas fluctuating regions are found among the residues ranging between 81–89 and 56–61. Each superimposed protein is shown in a different color (WNT-1: yellow, WNT-2B: magenta, WNT-6: blue, WNT-10A: red and WNT-10B: green).

**Figure 4:**
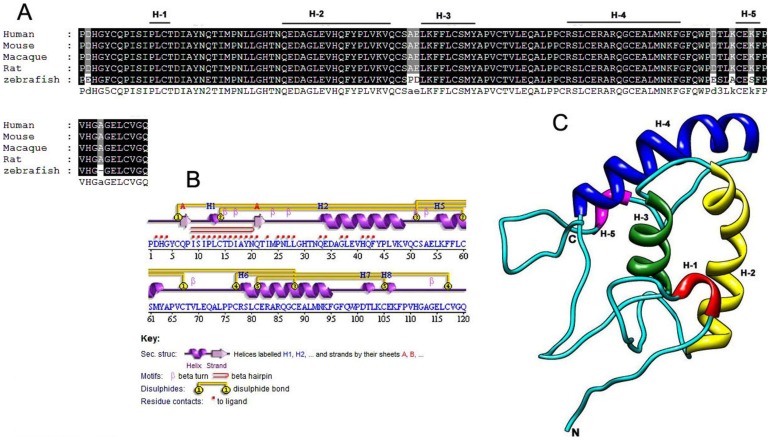
Multiple sequence alignment of orthologous cysteine rich domain. (A) Conservation of a Frizzled-1 CRD sequence in Human compared with the sequences of Macaque, Mouse, Rat and Zebra-fish. Except a few variations in zebra-fish sequence, the rest of the sequences of CRD are 100% identical. The multiple sequence alignment is generated by ClustalX and labeled by GenDoc. It also contains the important secondary structures elements. **(B)** Secondary structure of FZD-1 CRD. The secondary elements are predicted by PSIPRED. The strong extensive disulphide bridge formation is the most important feature of CRD. Additionally, beta hairpin motif and important residues for ligand binding are also predicted by PSIPRED. **(C)** Homology modeled three dimensional structure of CRD. Structure is built by Modeller9v8 using mouse CRD as a template (1IJY). The RMSD value calculated after superimposition was 0.62 Å.

The overall RMSD values for the predicted models of WNT backbone range from 0.3 to 0.6 Å. Evolutionarily highly conserved backbone structure were observed in all models with few fluctuating residues and rotamers. In **Fig. 3**, residues between 57 and 80 showed the conserved region, whereas small divergence was observed in case of Val-81- Gly-88 and Val-60- Ser-68. Comparing the sequences of CRD across various species, it was inferred that the domain's sequence is ∼99% conserved among vertebrates (human, macaque, mouse, rat and zebra-fish) (**Fig. 4A**). The secondary structure of CRD predicted by PSIPRED [31] showed extensive disulphide bridges, formed due to the presence of a high number of conserved cysteine residues. Along with this, many residues of CRD were predicted for ligand contacts which were further tested for interaction with WNT protein ligands during protein-protein interaction studies. However, the important predicted helices, strands and coils were also found in the three-dimensional modeled structure, using mouse CRD (1IJY) as a template (**Fig. 4B & 4C**). Possible topologies of secondary structures (Figure S2, S3) in the folded protein were predicted by pdbsum [32]. The overall RMSD value calculated for human CRD-1 against mouse CRD-8 was 0.62 Å.

### Prediction of CRD dimmer binding interactions

**Figure 5:**
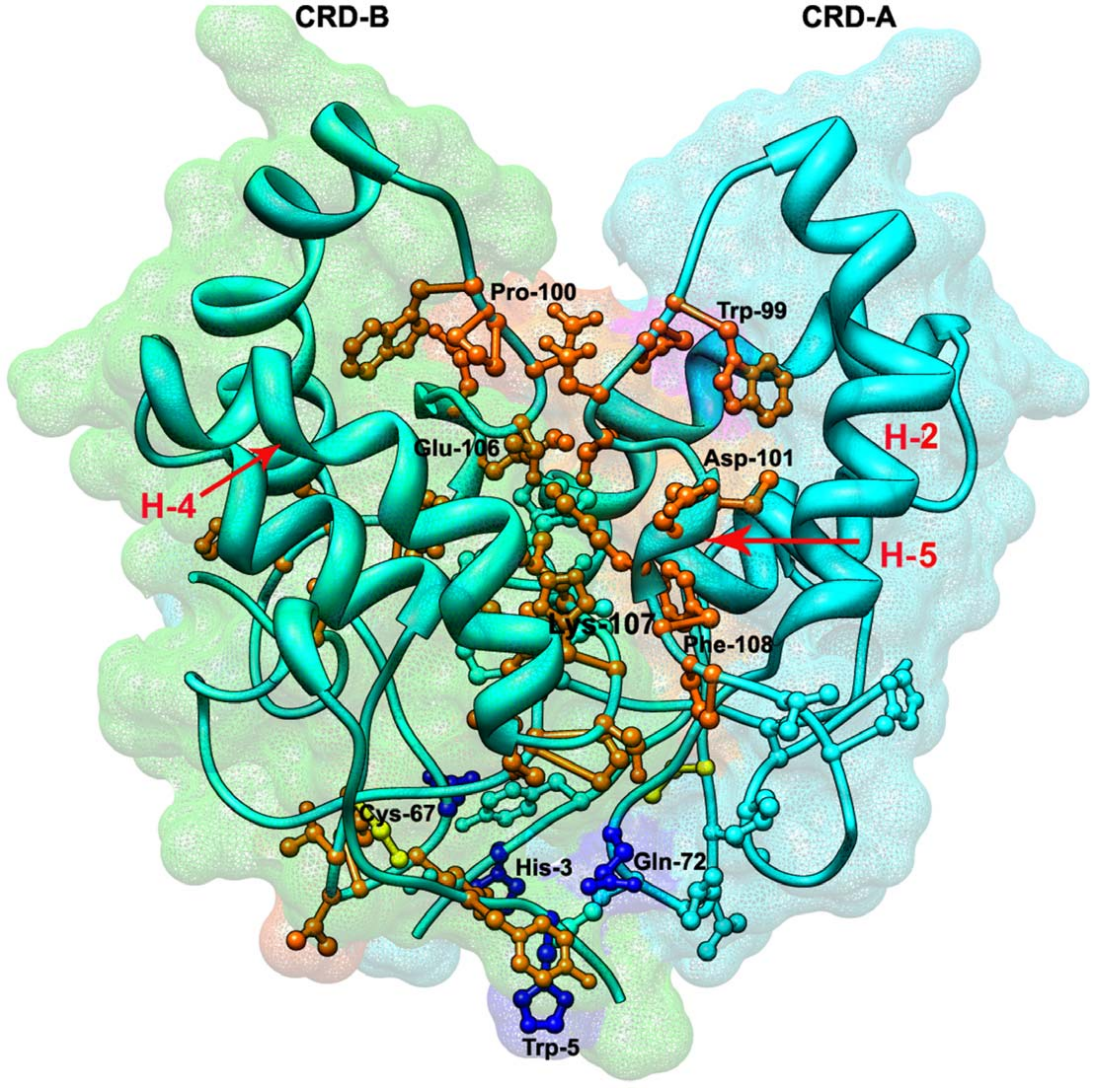
Predicted docked surfaces of CRD dimmer. The interacting residues are shown in different colors on the basis of their interactions. The residues involved in hydrogen bonding are shown in blue; residues connected to each other with disulphide bridge formation are shown in yellow, whereas the electro statically interacting residues are shown in orange red. This dimmer surface has the greatest interactions and has binding potential to bind with WNT ligands and hence could be targeted for inhibition. H-2 and H-4 helices and a loop region between these helices are involved in binding interactions.

Dann et al., 2001 [33] reported that the dimeric feature of CRD was important for WNT binding and may initiate the signaling pathway. Amino acid residues involved in dimerization of CRD can give us a strong structural insight about its mechanism of action and also enable us to trace important target residues for designing drug inhibitors against the WNT pathway. The minimized free energy CRD dimmer complex generated from protein-protein interaction was subjected to detailed analysis to study the electrostatics and van der waals interaction between residues of complex. The amino acid residues ranging from Pro-25 to Glu-39 showed strong hydrophobic interactions. The peptide fragment of both CRD (A & B), consisting of Cys-67, Thr-68, Val-69, Leu-70, Glu-71, Gln-72, Ala-73, Pro-75, were found to have strong hydrogen bonding, hydrophobic interactions and disulphide bridge formation **(** **Fig. 5** **)**. In total, three strong hydrogen bonding interactions were observed in dimmer formation. One of the hydrogens of the amide group of Gln-72 was found to bind with the oxygen of the carboxylic group of His-3, and the other interacted with the carboxylic group of Tyr-5. The third hydrogen bonded interaction was observed between Thr-102 (A) and Thr-102 (B). Both polar residues were found to have a strong bonded interaction with each other. Further electrostatic interactions were found within the loops of helices; H2, H5 and H4. The interaction was with residues Pro-100, Glu-106 and Lys-107 of H4 and Phe-106, Asp-101 and Trp-99 of H5 suppressed by H2 as shown in **Fig. 5** in detail.

### Binding interaction of WNT and CRD

**Figure 6:**
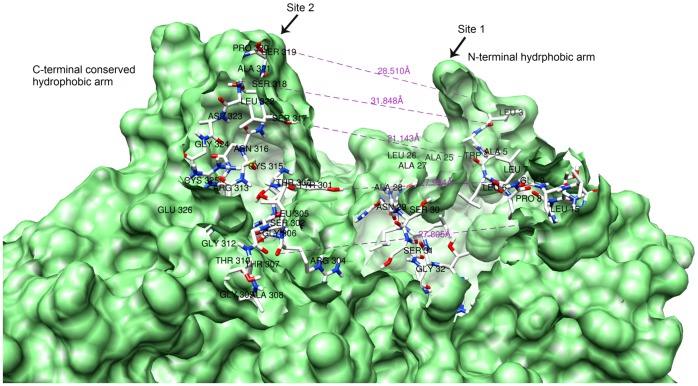
WNT U-shaped binding pocket with two hydrophobic arms grasping Frizzled CRD. N-terminal binding Site 1 contains most hydrophobic amino acid residues including Leu-3, Ala-5, and Pro-8. Binding site 2 is present at C-terminal end. This binding site was found to be most conserved in all WNT proteins, ranging between amino acid residues 300–325. The opening of the U-groove is 28 Å wide. The groove then widens to 31 Å, before narrowing to 17 Å at its centre and finally widening again to 27 Å at the base.

More than 30 amino acid residues were found to be engaged in binding of the WNT ligand with the CRD domain. The binding cleft forms a U-shaped contour into which the receptor fits and initiates the signaling cascade. The hydrophobic residues present at both arms of U-shaped pocket grasp the CRD domain by generating a strong hydrophobic moiety around it. Here we suggest two active binding sites of the WNT protein. Site 1 constitutes the right arm of the U-shaped pocket, consisting of N-terminal hydrophobic residues; the left C-terminal arm is site 2. A few residues at the base of the U-shaped groove also participate in binding interactions with Frizzled CRD. Measuring the atomic distances revealed that the opening face of the U-shaped groove is wide enough (starting at ∼28 Å then widening to ∼31 Å), to allow the receptor's CRD to fit in. The groove narrowed to ∼17 Å in the middle; however the base of the groove was found to be wide **(** **Fig. 6** **)**.

## Discussion

The chromosomal location of WNT proteins revealed that WNT gene clusters originated through block duplication events. WNT-1 and WNT-6 exist in the form of a cluster at chromosome 12 and 2q35 respectively and both are the closest homolog of each other. Therefore, in order to predict the structure of WNT-6, the WNT-1 modeled structure was used as a template. The sequence similarity between the two proteins is ∼42%; however, the model obtained for WNT-6 has low structural deviation and good quality factor values. The structures of the other homologous cluster of WNT-10A and WNT-10B were also generated computationally. The quality parameters of all modeled structures were verified including Q-score, Z-score, SDM, RMSD and DOPE plots.

The RMSD curve generally correlates with the percentage sequence identity, suggesting lower deviation values and strong structural homology between WNT proteins and their templates. However, some notable differences between the structures of template and target proteins were observed due to the insertions and deletions in loop regions during alignment. Most of the alignment errors occurred due to side-chain packaging, loop modeling and occasional alignment errors [34]. DOPE plots clearly supported the computationally modeled structures while measuring the residue by residue energy values. These energy plots proved the stability of modeled protein structures and can be processed for further analysis.

Molecular docking studies of CRD dimmer interaction and the WNT-CRD complex enabled us to locate the critical residues for targeted drug discovery. One of the approaches to inhibit the WNT pathway could be to target the amino acid residues involved in accurate processing of WNT ligands (mostly cysteines, N-glycosylated asparagines and O-glycosylated Serine and Threonine). After careful examination of all types of interactions (CRD-A with CRD-B and CRD with WNT), an interface summary of three proteins was drawn (Figure S4).

Two chains of CRD dimmer interacted with one another with the help of three alpha helices, labeled as H4 (B), H5 (A) and H2 (A) and loop region in between. Most of the amino acid residues present at the N-terminal end are involved in dimmer hydrophobic interactions. This dimmer then further fits itself into a U-shaped large groove serving as the WNT active binding pocket. Here we suggest Gln-72 as a very strong candidate to be targeted among dimmer interacting residues. Due to its strong and dual hydrogen binding behavior, it could serve as a very active target for pharmacophore drug modeling. Along with this, we also suggest a peptide fragment of Trp-99, Pro-100, Asp-101, Thr-102, Glu-106, Lys-107, Phe-108, Pro-109 and Leu-116, which forms a strong contour and hydrophobic pocket involved in dimmer interactions. Either by designing an inhibitor against a single residue or a whole peptide, we could stop the CRD dimmer interaction which could eventually stop the WNT signaling cascade.

Similarly, a U-shaped binding pocket was found to be conserved in all modeled WNT proteins. This conserved site is proposed to be one of the most important sites in WNT protein structure and is involved in the binding interaction with CRD. Our docking analysis suggested that this conserved binding groove could be targeted in order to design the inhibitors of the WNT signaling pathway. Interface summary of the WNT-CRD protein complex and binding interactions of WNT ligands with CRD allowed us to confirm the binding residues involved in both protein interactions. Most of the residues involved in N-glycosylation and cysteine rich region of WNT-1 (Table S2) were found to be actively participating in the binding interactions between CRD dimmer and WNT protein.

## Conclusion

The present structural study proposes a U-shaped binding contour in WNT protein structure for receiving the receptor's cysteine rich domain. This is strongly corroborated by Janda et al., 2012 [18] and Beinz, 2012 [35], who described the U-shaped groove as index finger and thumb grasping the CRD.

Along with this, our study proposes the evolutionarily conserved superimposed amino acid residues which could possibly be utilized in the targeted therapy of the WNT signaling pathway. The computationally predicted structures of the WNT protein give us a strong structural insight into initiation of WNT signaling cascade. Our study also addresses the problem of targeted therapy by locating the critical amino acid residues. These residues could help us to establish a pharmacophore model of novel drugs against the WNT pathway. Furthermore, our study supports the possible peptides suggested by Voronkov, 2008 [16] and predicts new interacting peptides in human WNT-1 and FZD-1 complex which could further be exploited for drug discovery.

## Supporting Information

Figure S1 Ramachandran Plot of CRD dimmer protein computationally modeled by homology modeling approach. All residues of the model lie within fully allowed, additionally allowed and generously allowed region except Ala 18-A. The Phi and Psi positions of the disallowed residue have been mentioned in the figure.
(TIF)

Figure S2 Secondary structure topology human CRD protein (chain A). Topology diagram depicts the residues involved in the formation of secondary structures elements (alpha helices and beta-sheets). These structures were created by PdbSum database.
(TIF)

Figure S3 Topology of human WNT protein. Topology diagram depicts the residues involved in the formation of secondary structures elements (alpha helices and beta-sheets). These structures were created by PdbSum database.
(TIF)

Figure S4 Interface summary of CRD dimmer and WNT interactions. The trimmer formed by the CRD dimmer protein and the WNT ligand contains three different types of interaction (hydrogen bonding: blue; electrostatic van der waal: orange; disulphide bridges: orange-red). The chains of CRD are labeled as A and B; WNT is labeled as C. The interface summary was created by PdbSum.
(TIF)

Table S1 Quality parameters of homology modeled three dimensional structures of proteins. The sequence similarity was calculated by ClustalX. Rotamer analysis, Ramachandran outliers and quality factor was measured by Molprobity server and NIH web server. For Q-score, SDM and RMSD values Chimera 1.5.3 was used.
(DOC)

Table S2 Functionally annotated sites of predicted models of WNT-1, WNT-6, WNT-10A and WNT-10B. N-glycosylation site is the most important site for post-translational modification of WNTs. Cysteine rich region at N-terminal end of the WNTs is important for cell signaling and lipid modification of WNTs.
(DOC)

## References

[1] MacDonald BT, Tamai K, He X (2009) Wnt/beta-catenin signaling: components, mechanisms and diseases. Dev Cell 17: 9–26. 19619488 10.1016/j.devcel.2009.06.016 PMC2861485

[2] Miller JR (2002) The Wnts. Genome Biol 3: 3001.1. 10.1186/gb-2001-3-1-reviews3001 PMC150458 11806834

[3] Iozzo RV, Eichstetter I, Danielson KG (1995) Aberrant expression of the growth factor Wnt-5A in human malignancy. Cancer Res 55: 3495–3499. 7627953

[4] Cadigan KM, Nusse R (1997) Wnt signaling: a common theme in animal development. Genes Dev 11: 3286–3305. 9407023 10.1101/gad.11.24.3286

[5] Kumar M, Ahmad S, Ahmad E, Saifi MA, Khan RH (2012) *In-Silico* prediction and analysis of Caenorhabditis EF-hand containing proteins. PLoS ONE 7: e36770. 22701514 10.1371/journal.pone.0036770 PMC3360750

[6] Satoh S, Daigo Y, Furukawa Y, Kato T, Miwa N, et al (2000) AXIN1 mutations in heptocellular carcinomas, and growth suppression in cancer cells by virus-mediated transfer of AXIN1. Nat Genet 24: 245–250. 10700176 10.1038/73448

[7] Sunaga N, Kohno T, Kolligs FT, Fearon ER, Saito R, et al (2001) Constitutive activation of the Wnt signaling pathway by CTNNB1 (beta-catenin) mutations in a subset of human lung adenocarcinoma. Genes Chromosomes Canc 30: 316–321. 10.1002/1098-2264(2000)9999:9999<::aid-gcc1097>3.0.co;2-9 11170292

[8] Holcombe RF, Marsh JL, Waterman ML, Lin F, Milovanic T, et al (2002) Expression of Wnt ligands and Frizzled receptors in colonic mucosa and in colon carcinoma. Mol Pathol 55: 220–226. 12147710 10.1136/mp.55.4.220 PMC1187182

[9] Akiyama T (2000) Wnt/β-catenin signaling. Cytokine Growth F R 11: 273–282. 10.1016/s1359-6101(00)00011-3 10959075

[10] Cong F, Schweitzer L, Varmus H (2004) Wnt signals across the plasma membrane to activate the beta-catenin pathway by forming oligomers containing its receptors, Frizzled and LRP. Development 131: 5103–5115. 15459103 10.1242/dev.01318

[11] Barker N, Clevers H (2006) Mining the Wnt pathway for cancer therapeutics. Nat Rev Drug Discov 5: 997–1014. 17139285 10.1038/nrd2154

[12] Mikels AJ, Nusse R (2006) Purified Wnt5a protein activates or inhibits beta-catenin-TCF signaling depending on receptor context. PLoS Biol 4: e115. 16602827 10.1371/journal.pbio.0040115 PMC1420652

[13] Malinauskas T (2008) Docking of fatty acids into the WIF domain of the human Wnt inhibitory factor-1. Lipids 43: 227–230. 18256869 10.1007/s11745-007-3144-3

[14] Malinauskas T (2011) Modular mechanism of Wnt signaling inhibition by Wnt inhibitory factor 1. Nat Struct Mol Biol 18: 886–893. 21743455 10.1038/nsmb.2081 PMC3430870

[15] Carron C, Pascal A, Djiane A, Boucaut JC, Shi DL, et al (2003) Frizzled receptor dimerization is sufficient to activate the Wnt/beta-catenin pathway. J Cell Sci 116: 2541–2550. 12734397 10.1242/jcs.00451

[16] Voronkov AE, Baskin II, Palyulin VA, Zefirov NS (2008) Molecular model of the Wnt protein binding site on the surface of dimeric CRD domain of the hFzd8 receptor. DoKl Biochem Biophys 419: 75–78. 18505162 10.1134/s1607672908020087

[17] Ain QU, Seemab U, Nawaz S, Rashid S (2011) Integrative analyses of conserved WNT clusters and their co-operative behavior in human breast cancer. Bioinformation 7: 339–346. 22355234 10.6026/97320630007339 PMC3280488

[18] Janda CY, Waghray D, Levin AM, Thomas C, Garcia KC (2012) Structural basis of Wnt recognition by Frizzled. Science 337: 59–64. 22653731 10.1126/science.1222879 PMC3577348

[19] Thompson JD (2002) Multiple sequence alignment using ClustalW and ClustalX. Curr Protoc Bioinformatics. John Wiley and Sons Inc. 10.1002/0471250953.bi0203s00 18792934

[20] Hubbard T, Barker D, Birney E, Cameron G, Chen Y, et al (2002) The Ensemble genome database project. Nucleic Acids Res 30: 38–41. 11752248 10.1093/nar/30.1.38 PMC99161

[21] Deshpande N, Addess KJ, Bluhm WF, Merino-Ott JC, Townsend-Merino W, et al (2005) The RCSB Protein Data Bank: a redesigned query system and relational database based on the mmCIF schema. Nucleic Acids Res 33: 233–237. 10.1093/nar/gki057 PMC540011 15608185

[22] Fiser A, Sali A (2003) Modeller: generation and refinement of homology-based protein structure models. Methods Enzymol 374: 461–491. 14696385 10.1016/S0076-6879(03)74020-8

[23] Pettersen EF, Goddard TD, Huang CC, Couch GS, Greenblatt DM, et al (2004) UCSF Chimera–a visualization system for exploratory research and analysis. J Comput Chem 25: 1605–1612. 15264254 10.1002/jcc.20084

[24] Laskowski RA, MacArthur MW, Moss DS, Thornton JM (1993) PROCHECK: a program to check the stereo chemical quality of protein structure. J Appl Cryst 26: 283–291.

[25] Gopalakrishnan K, Sowmiya G, Sheik SS, Sekar K (2007) Ramachandran plot on the web (2.0). Protein Pept Lett 14: 669–71. 17897092 10.2174/092986607781483912

[26] Emsley P, Cowtan K (2004) Coot: model-building tools for molecular graphics. Acta Crystallogr D 60: 2126–2132. 15572765 10.1107/S0907444904019158

[27] Krissinel E, Henrick K (2004) Secondary structure matching (SSM), a new tool for fast protein structure alignment in three dimensions. Acta Crystallogr D 60: 2256–2268. 15572779 10.1107/S0907444904026460

[28] Humphrey W, Dalke A, Schulten K (1996) VMD: Visual Molecular Dynamics. J Mol Graph 14: 33–38. 8744570 10.1016/0263-7855(96)00018-5

[29] Schneidman-Duhovny D, Inbar Y, Nussinov R, Wolfson HJ (2005) PatchDock and SymmDock: servers for rigid and symmetric docking. Nucleic Acids Res 33: W363–367. 15980490 10.1093/nar/gki481 PMC1160241

[30] Mashiach E, Schneidman-Duhovny D, Andrusier N, Nussinov R, Wolfson HJ (2008) FireDock: a web server for fast interaction refinement in molecular docking. Nucleic Acids Res 36: W229–232. 18424796 10.1093/nar/gkn186 PMC2447790

[31] McGuffin LJ, Bryson K, Jones DT (2000) The PSIPRED protein structure prediction server. Bioinformatics 16: 404–405. 10869041 10.1093/bioinformatics/16.4.404

[32] Laskowski RA, Hutchinson EG, Michie AD, Wallace AC, Jones ML, et al (1997) PDBsum: a Web-based database of summaries and analyses of all PDB structures. Trends Biochem Sci 22: 488–490. 9433130 10.1016/s0968-0004(97)01140-7

[33] Dann CE, Hsieh JC, Rattner A, Sharma D, Nathans J, et al (2001) Insights into Wnt binding and signaling from the structures of two Frizzled cysteine-rich domains. Nature 412: 86–90. 11452312 10.1038/35083601

[34] Baker D, Sali A (2001) Protein structure prediction and structural genomics. Science 294: 93–96. 11588250 10.1126/science.1065659

[35] Bienz M, He X (2012) Biochemistry. A lipid linchpin for Wnt-Fz docking. Science 337: 44. 22653728 10.1126/science.1224468

