# Supplementary figures and images for: Prediction of Structure of Human WNT-CRD (FZD) Complex for Computational Drug Repurposing
Source: PLoS One. 2013 Jan 25;8(1):e54630. doi: 10.1371/journal.pone.0054630 (PMC3556074; doi:10.1371/journal.pone.0054630)

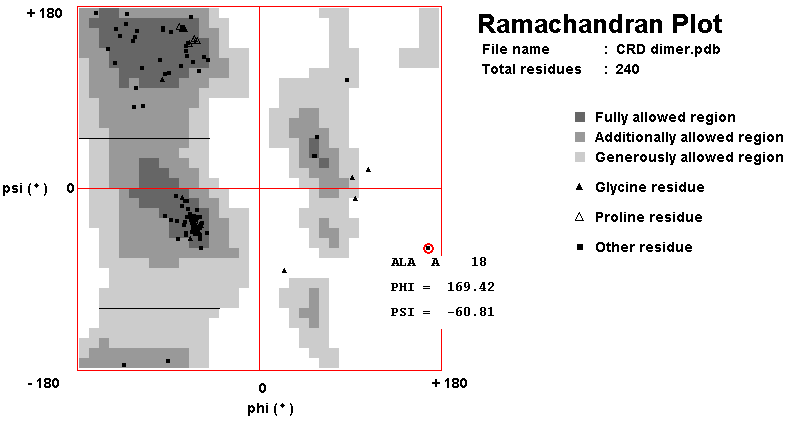

Supplement: Figure S1 — Ramachandran Plot of CRD dimmer protein computationally modeled by homology modeling approach. All residues of the model lie within fully allowed, additionally allowed and generously allowed region except Ala 18-A. The Phi and Psi positions of the disallowed residue have been mentioned in the figure. (TIF) [file pone.0054630.s001.tif]

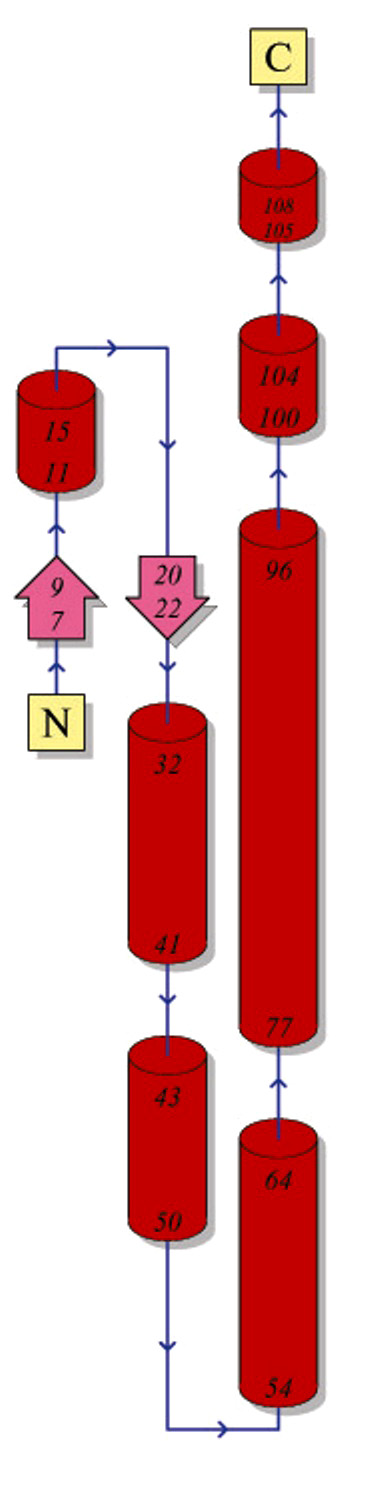

Supplement: Figure S2 — Secondary structure topology human CRD protein (chain A). Topology diagram depicts the residues involved in the formation of secondary structures elements (alpha helices and beta-sheets). These structures were created by PdbSum database. (TIF) [file pone.0054630.s002.tif]

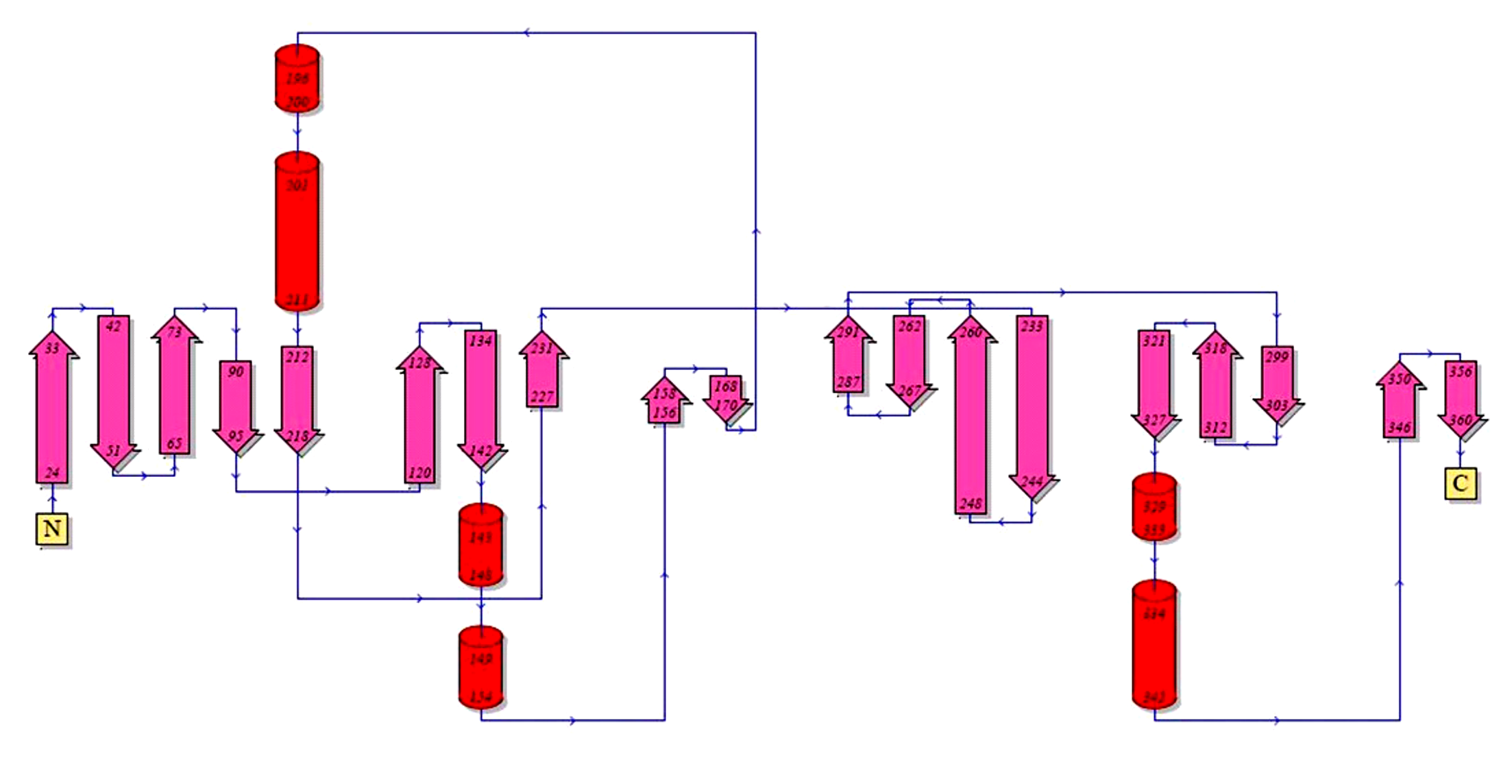

Supplement: Figure S3 — Topology of human WNT protein. Topology diagram depicts the residues involved in the formation of secondary structures elements (alpha helices and beta-sheets). These structures were created by PdbSum database. (TIF) [file pone.0054630.s003.tif]

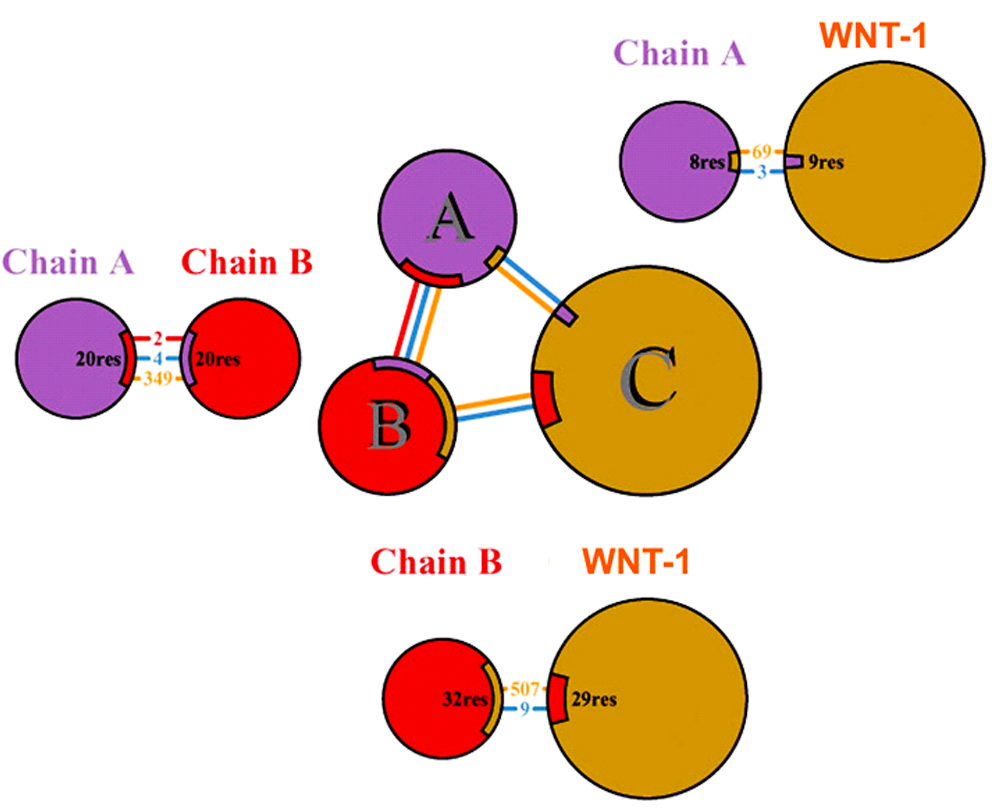

Supplement: Figure S4 — Interface summary of CRD dimmer and WNT interactions. The trimmer formed by the CRD dimmer protein and the WNT ligand contains three different types of interaction (hydrogen bonding: blue; electrostatic van der waal: orange; disulphide bridges: orange-red). The chains of CRD are labeled as A and B; WNT is labeled as C. The interface summary was created by PdbSum. (TIF) [file pone.0054630.s004.tif]
