# Supplementary material for: Prediction of Structure of Human WNT-CRD (FZD) Complex for Computational Drug Repurposing
Source: PLoS One. 2013 Jan 25;8(1):e54630. doi: 10.1371/journal.pone.0054630 (PMC3556074; doi:10.1371/journal.pone.0054630)
Supplement: Table S1 — Quality parameters of homology modeled three dimensional structures of proteins. The sequence similarity was calculated by ClustalX. Rotamer analysis, Ramachandran outliers and quality factor was measured by Molprobity server and NIH web server. For Q-score, SDM and RMSD values Chimera 1.5.3 was used. (DOC) [file pone.0054630.s005.doc]

| **Proteins** | | **Poor Rotamers** | **Ramachandran Plot** | **Overall Quality Factor** | **RMSD** | **Q-score** | **Average Z-score**  **mean** | **SDM** | **Template** | **Sequence similarity with template** |
| --- | --- | --- | --- | --- | --- | --- | --- | --- | --- | --- |
| FZD CRD | | 0 % | 95.34% | 78% | 0.62 | 0.881 | 2.35 | 12.44 | 1IJY | 50% |
| WNT-2B | | 0% | 92.54% | 48% | 0.8 | 0.77 | 2.21 | 15 | 1OLZ | 39% |
| WNT-1 | | 0% | 88.5% | 56.3% | 0.392 | 0.9 | 0.65 | 7.8 | WNT-2B | 40% |
| WNT-6 | | 0% | 89.2% | 50.5% | 0.53 | 0.89 | 0.8 | 10.9 | WNT-1 | 42% |
| WNT-10A | | 0% | 88% | 53.83% | 0.62 | 0.82 | 1.227 | 12.4 | WNT-6 | 42% |
| WNT-10B | | 0% | 90% | 48.46% | 0.4 | 0.8 | 0.448 | 9.2 | WNT-10A | 60% |
|  | **Table S1: Quality parameters of homology modeled three dimensional structures of proteins.** The sequence similarity was calculated by ClustalX. Rotamer analysis, Ramachandran outliers and quality factor was measured by Molprobity server and NIH web server. For Q-score, SDM and RMSD values Chimera 1.5.3 was used. | | | | | | | | | |
