# Supplementary material for: Prediction of Structure of Human WNT-CRD (FZD) Complex for Computational Drug Repurposing
Source: PLoS One. 2013 Jan 25;8(1):e54630. doi: 10.1371/journal.pone.0054630 (PMC3556074; doi:10.1371/journal.pone.0054630)
Supplement: Table S2 — Functionally annotated sites of predicted models of WNT-1, WNT-6, WNT-10A and WNT-10B. N-glycosylation site is the most important site for post-translational modification of WNTs. Cysteine rich region at N-terminal end of the WNTs is important for cell signaling and lipid modification of WNTs. (DOC) [file pone.0054630.s006.doc]

| **Functional Sites** | **WNT-1** | **WNT-6** | **WNT-10A** | **WNT-10B** |
| --- | --- | --- | --- | --- |
| WNT Family Signature | CKCHGMSGsC 218-227 | CKCHGMSGsC 222-231 | CKCHGTSGsC 262-271 | CKCHGTSGsC 247-256 |
| Cysteine Rich Region | CellCCgrghrtrtqrvterCnCtfhwCChvsCrnC 325-360 | CdllCCgrghrqesvqleenClCrfhwCCvvqChrC 320-355 | CgsmCCgrghnilrqtrserChCrfhwCCfvvCeeC 372-407 | CgslCCgrghnvlrqtrverChCrfhwCCyvlCdeC 344-379 |
| N-Glycosylation Site | NSSG 29-32 | NCSS 86-89 | NCSS 106-109 | NCSA 93-96 |
|  | NSSS 316-319 | NSSA 311-314 | NKSS 363-366 | NKTS 335-338 |
|  | NCTF 346-349 |  |  |  |
|  | NCTH 359-362 |  |  |  |
| Protein Kinase C Phosphorylation Sites | SGR 31-33 | TlK 275-277 | SrR 68-70 | SkR 55-57 |
|  | SRK 65-67 |  | SrR 162-164 | TSR 337-339 |
|  | TvR 288-230 |  | SeR 389-391 |  |
|  | TlR 238-240 |  |  |  |
|  | SnR 263-265 |  |  |  |
| N- Maristoylation Sites | GIlhSV 77-82 | GLwwAV 24-29 | GIqiAI 88-93 | GLHiAV 75-80 |
|  | GGlqSA 84-89 | GArlGV 68-73 | GVvhAV 140-145 | GVmhAV 127-132 |
|  | GLqSAV 85-90 | GVreCQ 72-77 | GCdaSR 158-163 | GTsgSC 251-256 |
|  | GNrGSN 259-264 | GAshAV 115-120 | GLshGV 187-192 | GSpgTR 325-330 |
| Casein Kinase II Phosphorylation Sites | ScSE 142-145 | SmgE 125-128 | SSlE 108-111 | SalE 95-98 |
|  | SraE 267-270 | TrtE 218-221 | SpaD 332-335 | TrVE 359-362 |
|  | SphD 285-288 | SapD 313-316 | TrSE 387-390 |  |
| Cell Attachment Sequence |  | RGD 195-197 | RGD 164-166 |  |
| cAMP- & cGMP-dependent protein kinase phosphorylation site |  |  | RRaS 329-332 | RRlS 302-305 |
| Tyrosine kinase phosphorylation site |  |  |  | RlsgElv.Y 303-310 |

**Table S2: Functionally annotated sites of predicted models of WNT-1, WNT-6, WNT-10A and WNT-10B.** N-glycosylation site is the most important site for post-translational modification of WNTs. Cysteine rich region at N-terminal end of the WNTs is important for cell signaling and lipid modification of WNTs.
